# New data on the valvatiform-shelled Hydrobiidae (Caenogastropoda, Truncatelloidea) from southern Greece

**DOI:** 10.3897/zookeys.1062.64746

**Published:** 2021-10-12

**Authors:** Sebastian Hofman, Jozef Grego, Zoltán Fehér, Zoltán Péter Erőss, Aleksandra Rysiewska, Artur Osikowski, Andrzej Falniowski

**Affiliations:** 1 Department of Comparative Anatomy, Institute of Zoology and Biomedical Research, Jagiellonian University, ul. Gronostajowa 9, 30-387 Kraków, Poland; 2 Horná Mičiná 219, SK-97401 Banská Bystrica, Slovakia; 3 WWF Hungary H-1141 Álmos vezér útja 69/a, Budapest, Hungary; 4 Levendula u. 68/4, H-2119, Pécel, Hungary; 5 Department of Malacology, Institute of Zoology and Biomedical Research, Jagiellonian University, ul. Gronostajowa 9, 30-387 Kraków, Poland; 6 Department of Animal Reproduction, Anatomy and Genomics, University of Agriculture in Krakow, al. Mickiewicza 24/28, 30-059 Kraków, Poland

**Keywords:** COI, H3, molecular phylogeny, new species, Peloponnese, spring gastropods

## Abstract

The minute valvatiform-shelled Hydrobiidae are less studied than other hydrobiid gastropods. In this paper, new data on these snails are presented, which have been collected at twelve springs in southern Greece: one in Boeotia, one on Evvoia Island, and ten on the Peloponnese Peninsula. Mitochondrial cytochrome oxidase subunit I (COI) and nuclear histone (H3) have been used to confirm the determinations and infer the relationships of the studied gastropods. They represent the genera *Daphniola*, *Graecoarganiella* and *Isimerope*. New localities, expanding the known geographic ranges, have been presented for *Daphniolahadei* and *Daphniolalouisi*. A species of *Daphniola* found at two localities has been identified as a species new to science, and its description, including the shell, penis, and female reproductive organs is given. Possible relationships between *Graecoarganiella* and *Isimerope* are discussed; their representatives are possibly new species. At one locality a single specimen likely represents a new genus: it was found to be most closely related with *Islamia*, but genetically (p-distance) too distant to be congeneric with *Islamia*.

## Introduction

Minute freshwater gastropods with depressed trochiform (valvatiform) shells often were classified as belonging to the family Valvatidae Gray, 1840. The first genus described for such hydrobiid snails was *Horatia* Bourguignat, 1887 from Dalmatia (Schütt 1961; Radoman 1965; Szarowska and Falniowski 2014). Hydrobiidae in Greece are still poorly studied, and their microhabitats have drastically disappeared (Szarowska and Falniowski 2011a). The poor knowledge is perhaps of more concern for the valvatiform-shelled hydrobiids, since their low-spired tiny shells are easily overlooked or treated as juveniles. Some authors, for example Schütt (1980), have expanded the ranges of the Central European and North Balkan genera towards Greece, which was criticized by for example, Radoman (1983, 1985). In the present paper, we present the valvatiform-shelled gastropods collected at 12 localities in southern Greece.

## Material and methods

**Figure 1.**
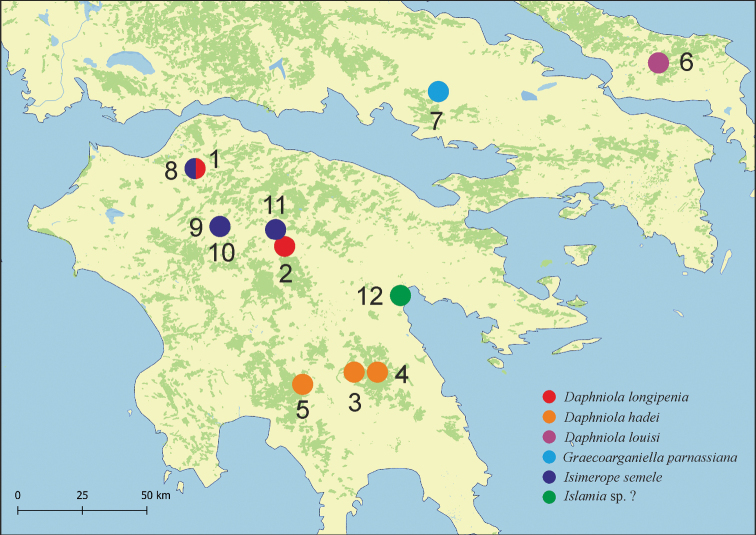
Localities of the sampling sites. For geographic coordinates see Table 1.

The snails were collected by hand or with a sieve at twelve localities listed in Table 1 (Fig. 1) during two trips in 2009 and 2018. Samples were sieved through 500 μm sieve and fixed in 80% analytically pure ethanol, replaced twice, and sorted later. Next, the snails were put in fresh 80% analytically pure ethanol and kept at -20 °C in a refrigerator. The shells were photographed with a Canon EOS 50D digital camera, under a Nikon SMZ18 microscope with dark field. Dissections were performed under a Nikon SMZ18 microscope with dark field, equipped with Nikon DS-5 digital camera. Captured images were used to draw anatomical structures with a graphic tablet. Morphometric parameters of the shell were measured all by the same person using a Nikon DS-5 digital camera and ImageJ image analysis software (Rueden et al. 2017).

**Table 1.** Geographic coordinates of identified sampling sites, by species. See also the map (Fig. 1). Extraction numbers (in bold) are also given, see also Figures 7, 8.

Id	Site	Coordinates
*Daphniolalongipenia*		
1	W edge of Katarraktis, spring 564 m, Achaia, Peloponnese, Greece; **2A29, 2B24**	38.1014, 21.8328
2	Panagitsa, large spring 500 m, Arcadia, Peloponnese, Greece; **2A32, 2B26, 2B27**	37.7725, 22.2219
*Daphniolahadei*		
3	3 km W of Megali Vrisi, Laconia, Peloponnese, Greece; **2A27**	37.2267, 22.5222
4	Spring beneath Katafigio Parnonos, Laconia, Peloponnese, Greece; **2B19**	37.2222, 22.6158
5	Kastorio, spring, 3 3 km N of village at aquaducte, Laconia, Peloponnese, Greece; **2B20**	37.1733, 22.2944
*Daphniolalouisi*		
6	Ag. Kiriaki spring N of Kato Kampia, Euboea, Greece; **2A33**	38.5608, 23.8442
*Graecoarganiellaparnassiana*		
7	mouth of Erkinas Gorge, Kria 2, Boeotia, Livadia, Greece **2A28, 2B23**	38.4319, 22.8750
*Isimeropesemele*		
8	Peloponnese, Achaia regional unit, Katarraktis center, spring and limetone cliffs, **2A30**	38.0989, 21.8342
9	Peloponnese, Achaia regional unit, Ag. Georgios (E of Tripotam), Vici spring, **2A31**	37.8525, 21.9397
10	Peloponnese, Achaia regional unit, Ag. Georgios (E of Tripotam), Anastasia spring, **2B21**	37.8517, 21.9408
11	Ladon spring E of Kerasia, Achaia regional unit, 474 m; 462 m alt., Greece, **2A22**	37.8361, 22.1819
cf. *Islamia* sp.		
12	Mili, spring below power station (on the Astros–Argos road), Argolis **2A34**	37.5525, 22.7175

Snails for molecular analysis were fixed in 80% ethanol, changed twice, and later stored in 96% ethanol. DNA was extracted from whole specimens; tissues were hydrated in TE buffer (3 × 10 min); then total genomic DNA was extracted with the SHERLOCK extraction kit (A&A Biotechnology), and the final product was dissolved in 20 μl of tris-EDTA (TE) buffer. The extracted DNA was stored at -80 °C at the Department of Malacology, Institute of Zoology and Biomedical Research, Jagiellonian University in Kraków (Poland).

Mitochondrial cytochrome oxidase subunit I (COI), and nuclear histone 3 (H3) loci were sequenced. Details of PCR conditions, primers used and sequencing were given in Szarowska et al. (2016). Sequences were initially aligned in the MUSCLE (Edgar 2004) program in MEGA 7 (Kumar et al. 2016) and then checked in BIOEDIT 7.1.3.0 (Hall 1999). Uncorrected p-distances were calculated in MEGA 7. Estimation of the proportion of invariant sites and the saturation test (Xia 2000; Xia et al. 2003) were performed using DAMBE (Xia 2013). In the phylogenetic analysis, additional sequences from GenBank were used as references (Table 2). The data were analysed using approaches based on Bayesian Inference (BI) and Maximum Likelihood (ML). We applied the GTR model whose parameters were estimated by RAxML (Stamatakis 2014). In the BI analysis, the GTR + I + Γ model of nucleotide substitution was applied. The model was selected using MrModelTest 2.3 (Nylander 2004). The Bayesian analyses were run using MrBayes v. 3.2.3 (Ronquist et al. 2012) with defaults of most priors. Two simultaneous analyses were performed, each with 10,000,000 generations, with one cold chain and three heated chains, starting from random trees and sampling the trees every 1000 generations. The first 25% of the trees were discarded as burn-in. The analyses were summarised as a 50% majority-rule tree. Convergence was checked in Tracer v. 1.5 (Rambaut and Drummond 2009). FigTree v. 1.4.4 (Rambaut 2010) was used to visualise the trees. The ML analysis was conducted in RAxML v. 8.2.12 (Stamatakis 2014) using the RAxML-HPC v.8 on XSEDE (8.2.12) tool via the CIPRES Science Gateway (Miller et al. 2010). Bootstrap support was calculated with 1000 replicates and summarised on the best ML tree.

**Table 2.** Taxa used for phylogenetic analyses (COI and H3) with their GenBank (GB) accession numbers and references.

Species	COIGB numbers	H3GB numbers	References
*Agrafiawiktori* Szarowska & Falniowski, 2011	JF906762	MG543158	Szarowska and Falniowski 2011b; Grego et al. 2017
*Alzoniellafinalina* Giusti & Bodon, 1984	AF367650	-	Wilke et al. 2001
*Anagastinazetavalis* (Radoman, 1973)	EF070616	-	Szarowska 2006
*Belgrandiellakuesteri* (Boeters, 1970)	MG551325	MG551366	Osikowski et al. 2018
*Dalmatinellafluviatilis* Radoman, 1973	KC344541	-	Falniowski and Szarowska 2013
*Daphnioladione* Radea, Lampri, Bakolitsas & Parmakelis, 2021	MW581160	-	Radea et al. 2021
*Daphniolaexiqua* (A. Schmidt, 1856)	EU047766, JF916470	-	Falniowski et al. 2007; Falniowski and Szarowska 2011a
*Dapniolagraeca* Radoman, 1973	EU047763	-	Falniowski et al. 2007
*Daphniolahadei* (Gittenberger, 1982)	JF916477, JF916479	-	Falniowski and Szarowska 2011a
*Daphniolahadei* (Gittenberger, 1982)	MZ093457- MZ093459	MZ265365–MZ265367	present study
*Daphniolalouisi* Falniowski & Szarowska, 2000	EU047769, KM887914, KM887915	-	Falniowski et al. 2007; Szarowska et al. 2014
*Daphniolalouisi* Falniowski & Szarowska, 2000	MZ093456	MZ265364	present study
*Daphniolalongipenia*	MZ093460- MZ093464	MZ265368–MZ265372	present study
*Daphniolamagdalenae* Falniowski, 2015	KT825578, KT825580	-	Falniowski and Sarbu 2015
*Ecrobiamaritima* (Milaschewitsch, 1916)	KX355835	MG551322	Osikowski et al. 2016/Grego et al. 2017
*Fissuriaboui* Boeters, 1981	AF367654	-	Wilke et al. 2001
*Grazianaalpestris* (Frauenfeld, 1863)	AF367641	-	Wilke et al. 2001
*Graecoarganiellaparnassiana* Falniowski & Szarowska, 2011	JN202349, JN202352	-	Falniowski and Szarowska 2011b
*Graecoarganiellaparnassiana* Falniowski & Szarowska, 2011	MZ093454- MZ093455	MZ265362–MZ265363	present study
*Grossuanaangeltsekovi* Glöer & Georgiev, 2009	KU201090	-	Falniowski et al. 2016
*Grossuanahohenackeri* (Küster, 1853)	KC011749	-	Falniowski et al. 2012
*Hauffeniamichleri* (Kuščer, 1932)	KT236156	KY087878	Falniowski and Szarowska 2015/Rysiewska et al. 2017
*Isimeropesemele* Radea & Parmakelis, 2013	KC841149	-	Radea et al. 2013
*Isimeropesemele* Radea & Parmakelis, 2013	MZ093450- MZ093453	MZ265358–MZ265361	present study
*Isimerope* sp.	JN202354		Falniowski and Szarowska 2011b
*Islamiazermanica* (Radoman, 1973)	KU662362	MG551320	Beran et al. 2016; Grego et al. 2017
*Islamia* sp.	MZ093465	MZ265373	present study
*Pontobelgrandiella* sp. Radoman, 1978	KU497024	MG551321	Rysiewska et al. 2016/Grego et al. 2017
*Radomaniolacurta* (Küster, 1853)	KC011814	-	Falniowski et al. 2012
*Sarajanaapfelbecki* (Brancsik, 1888)	MN031432	MN031438	Hofman et al. 2019

### Abbreviations

**GNHM**Goulandris Natural History Museum, Athens, Greece;

**HNHM**Hungarian Natural History Museum, Budapest, Hungary;

**JG** privat collection of Jozef Grego;

**ZMUJ**Zoological Museum of the Jagiellonian University, Kraków, Poland;

**ZPE** privat collection of Zoltán Péter Erőss.

## Results and discussion

### Systematics

**Family Hydrobiidae Stimpson, 1865**

#### Genus Daphniola

Taxon classificationAnimaliaLittorinimorphaHydrobiidae

Radoman, 1973

3E438A98-2AF9-5A6D-A77D-249D304DA297

##### Notes.

Radoman (1973) described this genus with its type species *D.graeca* Radoman, 1973, from the Daphne Spring in the valley of Tembe, North of Larissa. Schütt (1980) considered *D.graeca* a junior synonym of *Valvataexigua* Schmidt, 1856, described from “Greece”. Schütt (1980) designated a neotype from a group of small springs at Agia Paraskevi railway station, situated closely to the Daphne Spring, also in the valley of Tembe in Thessalia, but certainly not close to Thessaloniki as Kabat and Hershler (1993) state. Falniowski and Szarowska (2000) described *Daphniolalouisi* from a small spring at the monastery at Kessariani, Athens, Attica. The description was not considered by Bodon et al. (2001), who followed either Schütt (1980) in synonymizing *D.graeca* with *D.exigua*, or Reischütz and Sattmann (1993) in including Valvata (Cincinna) hellenica Westerlund, 1898 in *Daphniolaexigua*, thus rendering the genus *Daphniola* monotypic. Falniowski et al. (2007), applying soft-part morphology and anatomy as well as molecular markers, demonstrated the species distinctness of *D.louisi*, and identity of *D.exigua* with *D.graeca*. Gittenberger (1982) described *Horatiahadei*, a new species of *Horatia* he found 5 km SW of Yíthion (Gythion), southern Peloponnese. Later, Falniowski and Szarowska (2011a) collected this gastropod close to the (probably) destroyed type locality, and both, morphology and molecular data confirmed its classification within the genus *Daphniola*. Radea (2011) described *D.eptalophos* Radea, 2011 from the Parnassos Mountains. However, considering morphology, it is certainly not *Daphniola* especially in the intensively pigmented and massive penis. Its type locality is close (or rather the same) to the type locality of *Graecoarganiellaparnassiana* Falniowski & Szarowska, 2011. Thus “*Daphniolaetalophos*” is most probably a synonym of *Graecoarganiellaparnassiana*, and clearly belongs to *Graecoarganiella* rather than *Daphniola*. Szarowska et al. (2014) found a few juvenile specimens (used for DNA sequencing) of *Daphniola* sp. on each of the two Aegean islands: Rhodos and Khios. Falniowski and Sarbu (2015) described *D.magdalenae* Falniowski, 2015 from the sulphide Melissotripa Cave in Thessalia. Finally, Radea et al. (2021) described *D.dione* Radea, Lampri, Bakolitsas & Parmakelis, 2021 from the Levkas Island (Ionian Sea), using morphology and molecular data. At two localities in North Peloponnese (1 and 2) we found another *Daphniola* taxon, whose morphology and COI sequences do not comply with any known *Daphniola* species.

#### Daphniola longipenia

Taxon classificationAnimaliaLittorinimorphaHydrobiidae

Grego & Falniowski sp. nov.

9221FF57-AE8E-5CFF-9CE9-EFAD5DF8FD3C

http://zoobank.org/EE503BD0-65ED-4D53-AA37-ED75AB9448DA

##### Types.

**Figure 2.**
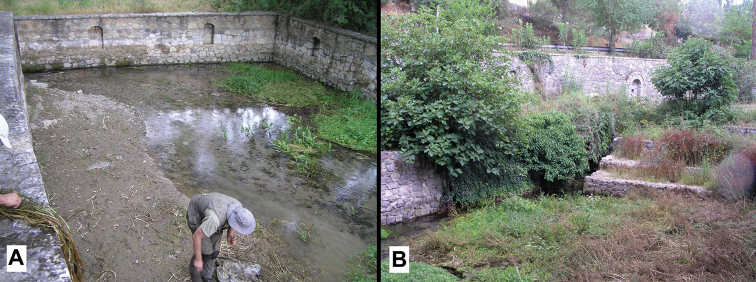
Type locality of *Daphniolalongipenia* sp. nov. Panagitsa, Arcadia, Peloponnese **A** spring reservoir **B** spring head.

Ethanol-fixed specimens, Panagitsa, large spring, Arcadia, Peloponnese, Greece, 37°46'21"N, 22°13'19"E (Fig. 2), altitude 500 m, sieved from sand at the spring head; 26.09.2009; Z.P. Eröss, Z. Fehér, T. Fehér, J. Grego and A. Hunyadi coll., ***holotype***: ZMUJ-M.2137; ***paratypes***: ZMUJ-M.2138-ZMUJ-M.2139, two paratypes; HNHM-105279, 10 wet and 25 dry paratypes, GNHM 39591, 10 paratypes, ZPE 25 dry paratypes, JG F1198, 11 wet and 72 dry paratypes.

##### GenBank numbers.

MZ093460–MZ093464; MZ265368–MZ265372

##### Diagnosis.

Shell minute, valvatiform-trochiform, soft parts slightly pigmented, penis with extremely long and slender filament and small non-glandular outgrowth (lobe) on the left side. Readily distinguished from *D.exigua*, *D.louisi*, *D.magdalenae* and *D.dione* by the proportionally much lower spire of the shell, and the penis with a narrower base and a longer and thinner filament. Differentiated from the geographically (but not molecularly) most close *D.hadei* by the shell with usually lower spire, and the penis with smaller outgrowth and still longer and thinner filament.

##### Description.

**Figure 3.**
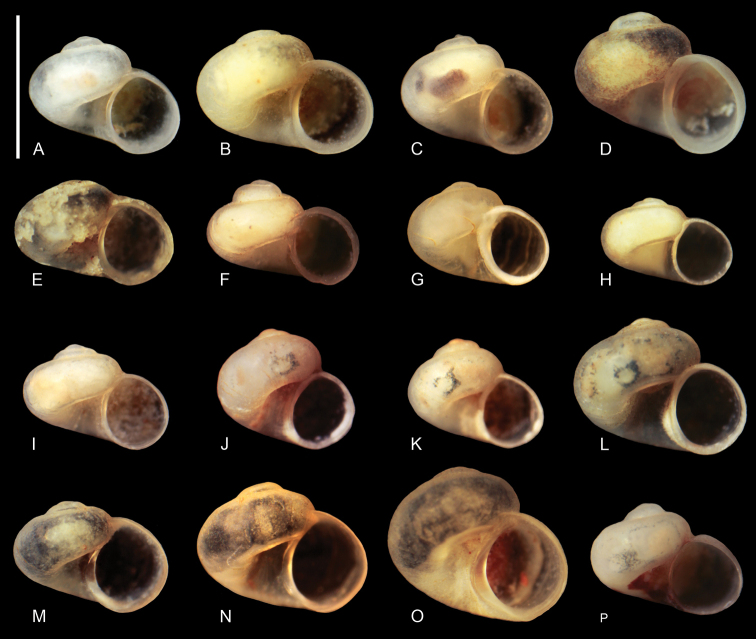
Shells of gastropods: *D.longipenia***A–D** locality 2 (holotype, 2A32, 2B26, 2B27) **E** locality 1 (2A29); *Daphniolahadei***F** locality 3 (2A27) **G** locality 4 (2B19) **H** locality 5 (2B20) **I***D.louisi*, locality 6 (2A33) **J–K***Graecoarganiellaparnassiana*, locality 7 (2A28, 2B23) **L–O***Isimerope***L** locality 8 (2A30) **M** locality 9 (2A31) **N** locality 10 (2B21) **O** locality 11 (2A22) **P** cf. *Islamia* sp., locality 12 (2A34). Scale bar: 1 mm.

Shell (Fig. 3A–E) valvatiform-trochiform, up to 1.00 mm tall, having 3.5 whorls, spire height 10–12% height of shell. Apex flat. Teleoconch whorls moderately convex, evenly rounded, growing rapidly in diameter. Aperture slightly elliptical, parietal lip complete, umbilicus very broad, outer lip simple, orthocline. Teleoconch with delicate growth lines, periostracum pinkish or yellowish. Shell parameters for holotype and a series of paratypes are given in Table 3. Inner and outer sides of operculum smooth. Operculum pinkish. Animal brownish, with some spots of black pigment.

**Table 3.** Shell measurements of *Daphniolalongipenia*; specimen symbols as in Figure 3; measured variables: see Figure 6.

	*a*	*b*	*c*	*d*	*e*	*a*
**A** – **holotype**	**0.87**	**0.75**	**0.60**	**0.12**	**0.54**	**121**
B – 2A32	0.88	0.82	0.66	0.10	0.57	127
C – 2B26	0.84	0.72	0.60	0.12	0.48	120
D – 2B27	1.00	0.82	0.66	0.14	0.57	121
E – 2A29	0.74	0.68	0.57	0.10	0.53	127
*M*	0.866	0.758	0.618	0.116	0.538	123.200
*SD*	0.093	0.062	0.040	0.017	0.037	3.493
*MIN*	0.74	0.68	0.57	0.10	0.48	120
*MAX*	1.00	0.82	0.66	0.14	0.57	127

**Figure 4.**
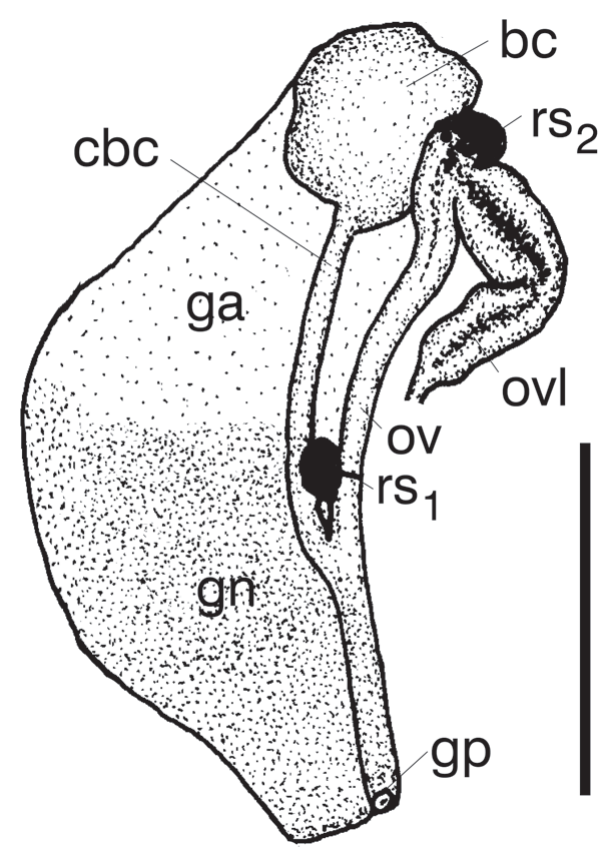
Pallial and renal section of female reproductive organs of *Daphniolalongipenia* [bc – bursa copulatrix, cbc – duct of bursa, ga – albuminoid gland, gn – nidamental gland, gp – gonoporus, ov – oviduct, ovl – loop of (renal) oviduct, rs – seminal receptacles (in black) rs_1_ and rs_2_ (as defined by Radoman 1973, 1983): rs_1_ – distal, rs_2_ – proximal]. Scale bar: 250 μm.

**Figure 5.**
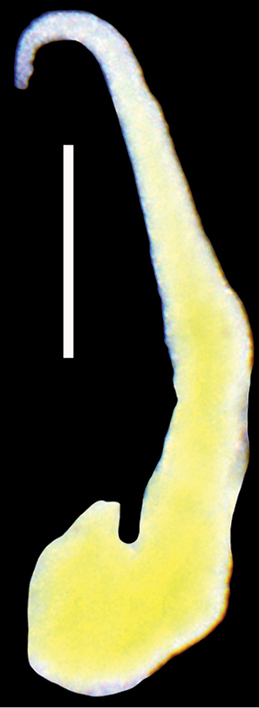
Penis of *Daphniolalongipenia*, bar equals 200 μm.

Female reproductive organs (Fig. 4) with a broad loop of the oviduct, a big bursa copulatrix with a long duct, and two moderately small receptacula seminis. Penis (Fig. 5) extremely long and narrow, simple, with an almost vestigial outgrowth proximally on its left edge, and a prominent sharp-terminated filament, vas deferens not visible inside.

##### Derivatio nominis.

The specific epithet *longipenia* refers to the extremely long filament of the penis.

##### Distribution and habitat.

Apart from the type locality (our locality 2), this species was also found in the spring at the W edge of Katarraktis, Achaia, Peloponnese (our locality 1).

##### Remarks.

**Figure 6.**
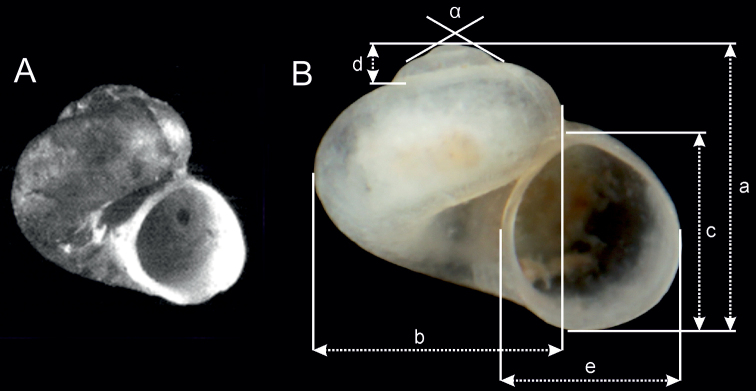
**A** shell of lectotype of *Valvatahellenica* presented by Reischütz and Sattmann (1993)**B** shell measurements: a – shell height, b – body whorl breadth, c – aperture height, d – spire height, e – aperture breadth, α – apex angle.

Westerlund (1898) described Valvata (Cincinna) hellenica Westerlund, 1898, from “Vyteria in Arkadien”. Reischütz and Reischütz (2004) identified Westerlund’s “Vyteria” as Vitina, situated about 15 km from Panagitsa. They reported *Hauffeniahellenica* (Westerlund, 1898) also from Panagitsa spring. Our *D.longipenia* is most probably the gastropod reported by them. However, their identification of “Vyteria” as Vitina remains doubtful. The shell of the lectotype of *Valvatahellenica* presented by Reischütz and Sattmann (1993) looks different (Fig. 6) (enormously high and massive body whorl, another size and outline of the aperture). *Valvatahellenica* was reported several times from localities scattered throughout Greece, often in generic combination with *Hauffenia* or *Daphniola*. It can be assumed that these records report more than one species; or it was mentioned as a younger synonym under *Daphniolaexigua* (e.g., Bodon et al. 2001). Summarising, the description of a new species is the most appropriate solution.

**Figure 7.**
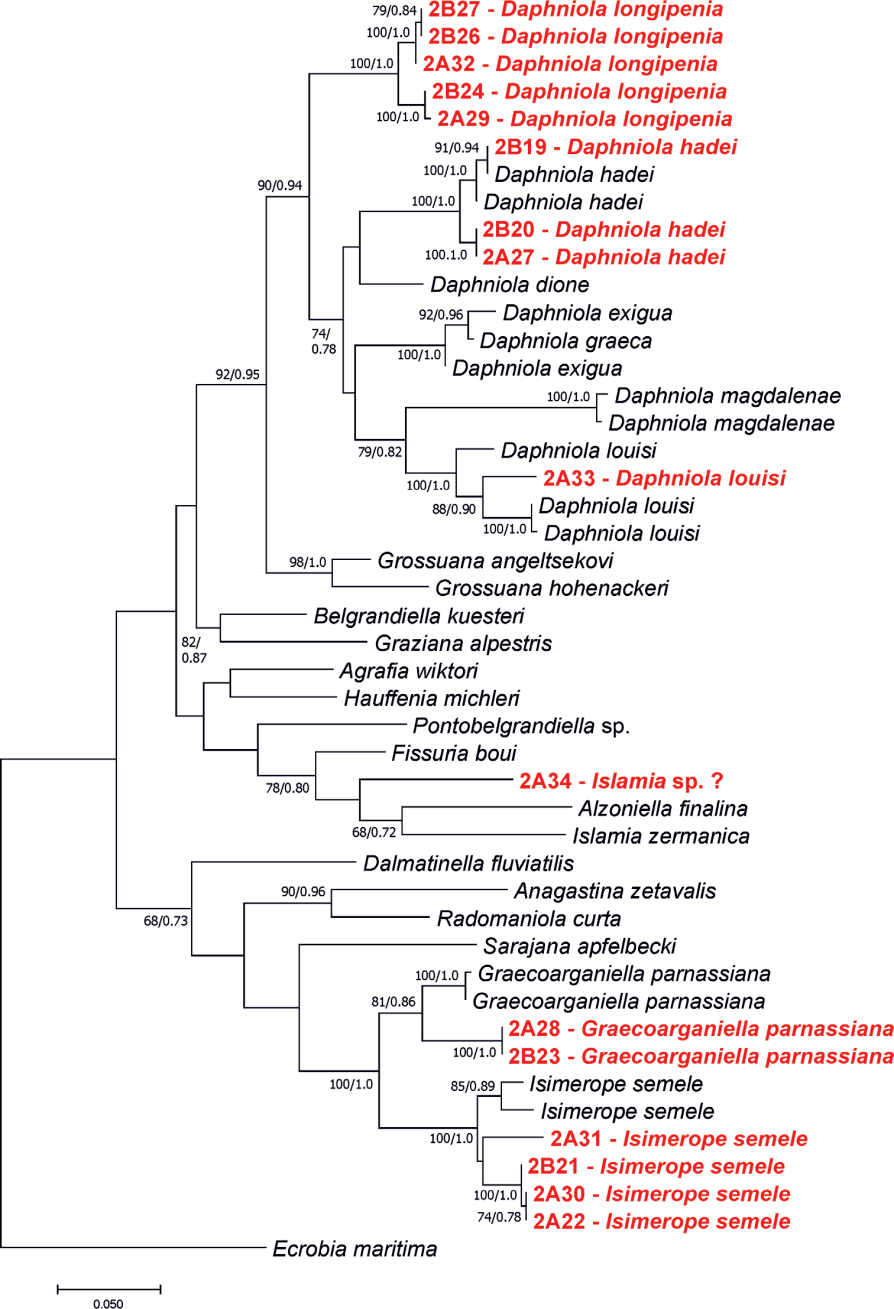
Phylogenetic tree for COI showing relationships between the studied snails. Bootstrap supports (>60%) and Bayesian probabilities are given.

**Figure 8.**
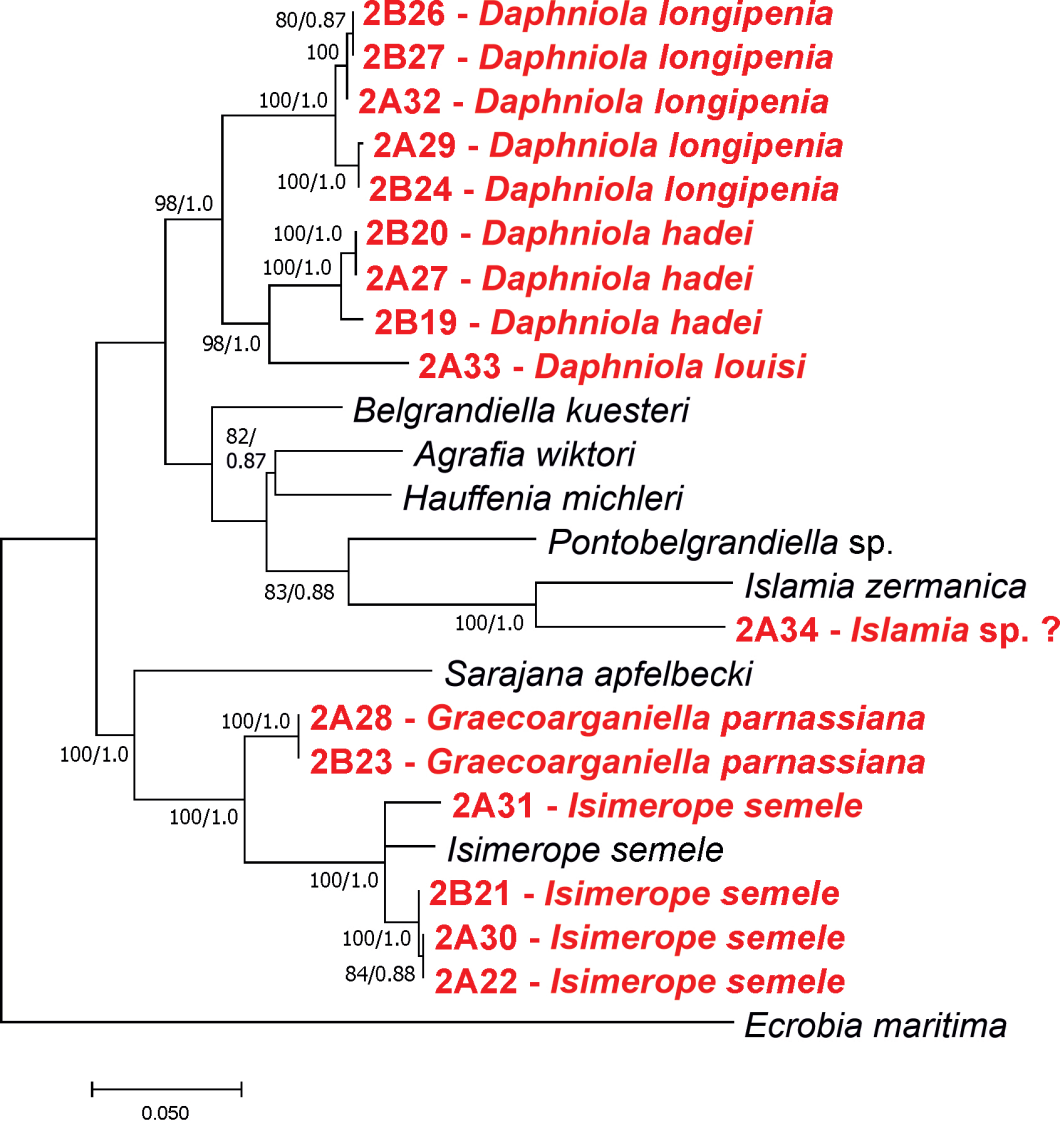
Phylogenetic tree inferred from connected COI and H3 sequences. Bootstrap supports (>60%) and Bayesian probabilities are given.

In our trees (Figs 7, 8), *D.longipenia* is clearly distinct from all the other species of *Daphniola* and forms a distinct sister clade opposite to the remaining currently known *Daphniola* species. The high genetic distance (p-distance 0.106) can be found between *D.longipenia* and *D.hadei* (p-distance 0.106), whose localities are most close, and who share the most similar shell morphology, female reproductive organs and penis). In general, the genetic distances between *D.longipenia* and the other *Daphniola* varies from 0.097 (for *D.exigua*) to 0.141 (for *D.magdalenae*) (Table 4).

**Table 4.** P-distances for COI between main clades of the *Daphniola*.

	*D.longipenia*	*D.hadei*	*D.dione*	*D.exiqua*	*D.magdalenae*
*D.longipenia*	–				
*D.hadei*	0.105	–			
*D.dione*	0.088	0.080	–		
*D.exiqua*	0.097	0.092	0.087	–	
*D.magdalenae*	0.141	0.153	0.133	0.154	–
*D.louisi*	0.121	0.103	0.097	0.110	0.122

#### Daphniola hadei

Taxon classificationAnimaliaLittorinimorphaHydrobiidae

(Gittenberger, 1982)

931FDF52-F105-51A3-9953-61EAB63A3050

##### GenBank numbers.

MZ093457–MZ093459; MZ265365–MZ265367

##### Notes.

At the localities 3, 4 and 5 (Fig. 1, Table 1) gastropods were collected, whose shells (Fig. 3F–H), soft parts morphology and anatomy, clearly identified them as belonging to this species. Their molecular data (Figs 7, 8) were identical or nearly identical with the ones published in the GenBank. Their localities are situated somewhat north of the type locality.

#### Daphniola louisi

Taxon classificationAnimaliaLittorinimorphaHydrobiidae

Falniowski & Szarowska, 2000

3C2EA04D-B370-5CCE-83A6-A81AEEC00C9D

##### GenBank numbers.

MZ093456, MZ265364

##### Notes.

The shell morphology (Fig. 3I), soft-part anatomy and molecular data (Figs 7, 8) of the gastropods collected at locality 6 (Fig. 1, Table 1), all showed that they belonged to this species. It has to be noted that the molecular variability in this species (p-distance 0.044) (Fig. 7) is much larger than in *D.hadei* (p-distance 0.013). The new record of *D.louisi*, located on Evvoia Island, considerably expands the range of the species, which so far was only known from Attica. The close phylogenetic relationship with the two juvenile *Daphniola* specimens collected on the Aegean islands, Rhodos and Khios, now combined with the present record from Evvoia, seems to confirm the ideas about the means of dispersal of *Daphniola* from continental Greece to the Aegean islands (Szarowska et al. 2014).

###### Genus *Graecoarganiella* Falniowski & Szarowska, 2011

#### Graecoarganiella parnassiana

Taxon classificationAnimaliaLittorinimorphaHydrobiidae

Falniowski & Szarowska, 2011

F4D207B6-180A-5172-8A8B-CC8B911E24F6

##### GenBank numbers.

##### Notes.

Falniowski and Szarowska (2011b) described a new, so far monotypic, genus of Hydrobiidae from Greece, Parnassus Mountains, S of Eptalofos, N of Kalania, found in a cistern and a small spring in a grassy pasture on a mountain pass. The type species, *G.parnassiana* Falniowski & Szarowska, 2011, is so far known only from the type locality. At the locality 7 (Fig. 1, Table 1), mouth of Erkinas Gorge, Kria 2, Boeotia, Livadia, we found gastropods, whose shells (Fig. 3J–K), and soft-part morphology indicated they belonged to *Graecoarganiella*, and were practically identical to *G.parnassiana*. Anatomy was not studied since the material was scarce and not fixed well enough. Our locality 7 is not far (about 35 km) from the type locality of *G.parnassiana*. The molecular data – partial sequences of COI – of our population showed rather high distinctness (Fig. 7). However, as can be seen in the same phylogram, these differences (p-distance 0.038) are a little lower than the ones within *Daphniolalouisi* (0.044). Thus, inclusion of our new population in *Graecoarganiellaparnassiana* is seemingly justified.

###### Genus *Isimerope* Radea & Parmakelis, 2013

#### Isimerope semele

Taxon classificationAnimaliaLittorinimorphaHydrobiidae

Radea & Parmakelis, 2013

E0994B43-FDC0-54EF-8712-CDC6661B4661

##### GenBank numbers.

MZ093450–MZ093453; MZ265358–MZ265361

##### Notes.

When describing *Graecoarganiellaparnassiana* from the Parnassus Mts., Falniowski and Szarowska (2011b) reported three young hydrobiid specimens found at Mainalo Mountain, Peloponnese, WSW of Piana, WNW of Tripolis, in a medium-sized spring and cistern. Their COI sequence was interpreted as indicating a distinct species congeneric with *Graecoarganiellaparnassiana*. Later, Radea et al. (2013) found other species at Megali Vrisi, Pharmakas Mt., and described it as a representative of a new monotypic genus *Isimerope*, with *I.semele* as the type species. In our tree (Fig. 7) *Graecoarganiella* and *Isimerope* are quite distinct (p-distance 0.096), but form a well-supported clade (bootstrap value of 100%, Bayesian probability 1.0). The shells are very similar, and the same holds true for the radulae. The lack of a ctenidium, and egg capsules laid in the umbilicus of the shell, might be considered as unique shared character states. The penes and female reproductive organs of the compared taxa do not differ more than could be expected by different season of collection or fixation technique.

At the four localities: 8, 9, 10 and 11 (Fig. 1, Table 1) we collected gastropods, whose shells (Fig. 3L–O), soft parts morphology (not well-fixed material reduced the possible examination) and molecular data (Fig. 7) showed them as belonging to *Isimerope*. Again, as in the case of *Graecoarganiella*, our specimens of *Isimerope* may represent distinct species, but as in *Daphniola*, the molecular differences may be considered as within- species level variation (p-distance 0.035).

Our molecular data clearly show the close relationship of *Isimerope* and *Graecoarganiella*, contradicting their classification to different subfamilies (Belgrandiinae de Stefani, 1877 and Horatiinae D. W. Taylor, 1966, respectively), as stated in WORMS (WoRMS Editorial Board 2021). Both more anatomical and molecular data, as well as a broad-scale revision of the systematics of the Truncatelloidea proposed by Bouchet et al. (2017) are badly needed.

#### cf. Islamia

Taxon classificationAnimaliaLittorinimorphaHydrobiidae

sp.

040CA6AF-C00A-5F50-80A0-ACC897B1F34F

##### GenBank numbers.

MZ093465; MZ265373

##### Notes.

At the locality 12, in Mili, Argolis, in a spring below the power station, a gastropod was found (Fig. 3P), whose molecularly inferred phylogenetic position (Fig. 8) remains enigmatic. Its sister taxon is *Islamia* Radoman, 1973. The clade’s bootstrap support for two concatenated loci is 100%, strongly suggesting that both mOTUs belong to the same taxon, but the genetic distance between them seems too high (p-distance 0.135). The p-distances in COI were 0.109 and 0.138 between this taxon and *Fissuria* Boeters, 1981 and *Alzoniella* Giusti & Bodon, 1984, respectively, although the shell morphology still suggests an affiliation with *Islamia*. Anyway, with only one more shell and lack of molecular data on the other Greek *Islamia* species, a justified taxonomic decision has to be postponed until more, and better, preserved specimens are available.

## Supplementary Material

XML Treatment for Daphniola

XML Treatment for Daphniola longipenia

XML Treatment for Daphniola hadei

XML Treatment for Daphniola louisi

XML Treatment for Graecoarganiella parnassiana

XML Treatment for Isimerope semele

XML Treatment for Islamia
